# The Impact of Polyphenols-Based Diet on the Inflammatory Profile in COVID-19 Elderly and Obese Patients

**DOI:** 10.3389/fphys.2020.612268

**Published:** 2021-01-12

**Authors:** Juliana Carvalho Santos, Marcelo Lima Ribeiro, Alessandra Gambero

**Affiliations:** ^1^ Lymphoma Translational Group, Josep Carreras Leukaemia Research Institute (IJC), Badalona, Spain; ^2^ Laboratory of Immunopharmacology and Molecular Biology, Sao Francisco University, Bragança Paulista, Brazil; ^3^ Life Science Center, Pontifical Catholic University of Campinas (PUCCAMP), Campinas, Brazil

**Keywords:** COVID-19, cytokine storm, inflammation, senescence, polyphenols

## Abstract

The World Health Organization declared the severe acute respiratory syndrome coronavirus-2 (SARS-CoV-2)-associated disease (coronavirus disease 2019 – COVID-19) as a pandemic in March 2020. COVID-19 is characterized by cytokine storm, acute respiratory distress syndrome (ARDS), and systemic inflammation–related pathology and already kills more than 1.5 million of people worldwide. Since aged and obese COVID-19 patients exhibit an enhanced inflammatory status, they represent a high-risk cluster for rapidly progressive clinical deterioration. These individuals present comorbid disorders and immunosenescence that may promote viral-induced cytokine storm and expression of molecules acting as virus receptor as angiotensin I converting enzyme 2 (ACE2) and CD26 (dipeptidyl-peptidase 4), resulting in respiratory failure and increased morbidity and mortality. A better knowledge of SARS-CoV-2 infection in inflammatory-associated high-risk population is essential in order to develop the therapies needed to combat or prevent severe COVID-19. Here, we review the pathogenesis and clinical implications of inflammatory disorders and disease markers associated to senescence in COVID-19 patients and the emerging evidence to argue that a high intake of polyphenols may have a protective effect on SARS-CoV-2 illness severity.

## Introduction

According to the World Health Organization, as of December 1st coronavirus disease 2019 (COVID-19) had been confirmed in almost 63 million of people worldwide, carrying a mortality of approximately 2.5%, with the vast majority of them (74%) being in people over 65 years (Webmeter, 2020; World Health Organization, 2020). Indeed, age is undoubtedly the most important risk factor for death in COVID-19 patients (Williamson et al., 2020). In addition, it has been reported that the severity of COVID-19 is associated with several comorbidities (i.e., respiratory system diseases, hypertension, diabetes, obesity, and cardiovascular disease; Webmeter, 2020). Although around 80% of confirmed severe acute respiratory syndrome coronavirus-2 (SARS-CoV-2) positive cases exhibit mild symptoms or are asymptomatic, the remaining 20% of patients may develop serious symptoms, potentially leading to death (Lai et al., 2020). These patients do not develop severe clinical manifestations in the early stages of the disease; however, an acute respiratory distress syndrome (ARDS) and multiple-organ failure can occur at later stages. Remarkably, it has been reported that respiratory failure is responsible for 86% of death associated to SARS-CoV-2 infection (Ruan et al., 2020).

The so-called cytokine storm has been pointed out as one of the major player in the process of disease aggravation (Chousterman et al., 2017; Shimabukuro-Vornhagen et al., 2018). Accordingly, *in vitro* data showed that a delayed release of cytokines and chemokines occurs in respiratory epithelial cells, dendritic cells, and macrophages at the early stage of SARS-CoV-2 infection. These cells secrete low levels of interferons (IFNs) and high levels of pro-inflammatory cytokines and chemokines (Cheung et al., 2005; Law et al., 2005; Lau et al., 2013) which attracts inflammatory cells, such as neutrophils and monocytes, resulting in excessive infiltration of the inflammatory cells into lung tissue, an consequent lung injury.

Cellular senescence is a conserved mechanism characterized by cell cycle arrest in response to both, extrinsic and intrinsic stimulation. Although senescent cells no longer replicate, they remain metabolically active and become bigger than non-senescent cells, secrete high levels of inflammatory proteins as part of the senescence associated secretory phenotype (SASP), and acquire cell metabolism changes (van Deursen, 2014). It has been shown that senescent cells may contribute to cell proliferation, inflammation (Freund et al., 2010), angiogenesis (Coppe et al., 2006), epithelial-to-mesenchymal transition (EMT; Laberge et al., 2012), and wound healing (Jun and Lau, 2010). Importantly, cellular senescence is also associated to age-related organ dysfunction and various chronic age-related diseases, such as Alzheimer, atherosclerosis, osteoarthritis, and pulmonary fibrosis (Naylor et al., 2013). Additionally, aging and most of age-related diseases are also related to a chronic systemic condition of inflammation, known as inflammageing (Michaud et al., 2013; Sanada et al., 2018).

The activation of immune system is another important source of chronic inflammation in virus-infected patients. Patients with COVID-19 harbor high levels of inflammatory cytokines, which may activate the T-helper type 1 (Th1) cell response (Huang et al., 2020). The host inflammatory response is driven by binding to toll-like receptors (TLRs), which recognize structural components belonging to viruses, a process known as “pathogen-associated molecular patterns” (PAMPs; Janeway and Medzhitov, 2002). Moreover, neutrophil infiltration in the lungs of individuals infected by SARS-CoV-2 may result in the secretion of damage-associated molecular patterns (DAMPs), as a cell death signal following the viral invasion (Tang et al., 2012; Cicco et al., 2020).

Thus, it has been suggested that the disturbance of inflammatory homeostasis in elderly COVID-19 patients may play a pivotal role in the risk of a cytokine storm and subsequently ARDS, enhancing the mortality risk (Koelman et al., 2019; Williamson et al., 2020). In this review, we discuss the relationship between senescence markers, present in elderly and obese individuals, and the severity of COVID-19. We also highlight the possibility that dietary polyphenols could be beneficial for population most affected by COVID-19 by modifying these senescence markers (Zhou et al., 2020).

## Pathogenesis of SARS-Cov-2 in Inflammatory Comorbidities

In early February 2020, the Chinese Center for Disease Control and Prevention (CDC) reported a large viewpoint (including 72,314 cases) summarizing that the case fatality was 8.0% (312 of 3,918) in patients 70–79 years old and 14.8% in patients aged ≥80 years (208 of 1,408; Wu and McGoogan, 2020). With the expansion of the pandemic throughout the world, it has been widely reported that elderly and geriatric adults are among the highest risk population for death among COVID-19 patients (Covino et al., 2020; Imam et al., 2020; Nguyen et al., 2020; Wang et al., 2020). Indeed, several meta-analysis confirmed that SARS-CoV-2 infection causes the highest morbidity and mortality in patients aged >60 years (Hu et al., 2020; Huang et al., 2020; Wang et al., 2020; Wu et al., 2020; Zheng et al., 2020). The reason for worsening the disease severity may be attributed to the immunosenescence and inflammageing (Chung et al., 2019; Rabi et al., 2020). Moreover, some comorbidities associated with age as hypertension, diabetes, chronic respiratory diseases, dysregulation of immune response, and obesity have been associated with severe COVID-19 (Chen et al., 2020b).

Obesity has previously been associated with hospitalization due to viruses infection, such as influenza and coronavirus (van Kerkhove et al., 2011; Moser et al., 2019). In addition, severe obesity is a risk factor associated with fatalities in hospitalized patients (Louie et al., 2011; Cocoros et al., 2014). In this sense, growing evidences indicate that obesity is also an important risk factor for worst prognosis among COVID-19 patients. The driving hypothesis point out to the axis excess of adipose tissue and inflammation, which exacerbate the cytokine storm associated with virus infection, as described below. Accordingly, several meta-analysis were able to show that comparing with non-obese patients, obese COVID-19 patients have higher risk to die (Santos et al., 2018; Halvatsiotis et al., 2020; Smith et al., 2020; Zheng et al., 2020). In addition, it has been reported that obese aged patients are more likely to be admitted at UCI for ARDS, and have also higher risk for fatality (Caussy et al., 2020; Lighter et al., 2020).

**Figure 1:**
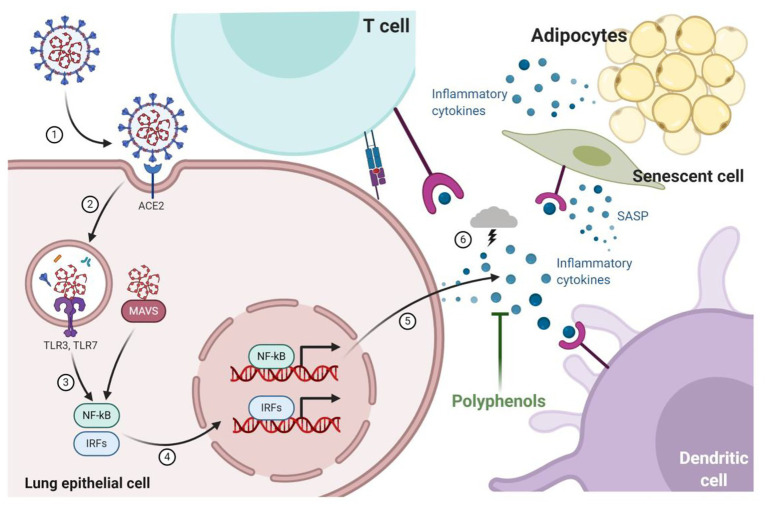
Infection of pulmonary epithelial cells occurs through the interaction of the spike glycoprotein envelope (S-protein) with the angiotensin I converting enzyme (ACE)-2 receptor that allows viral replication and triggers mechanisms to combat infection by the host cells thought toll-like receptors (TLRs) and mitochondrial antiviral-signaling protein (MAVS). Cytokines pro-inflammatory are produced by nuclear factor kappa -B (NF-kB) and interferon-regulatory factors (IRFs) signaling pathways recruiting more immune cells (dendritic cell and T-cell) to lungs. Recruited immune cells increased cytokine production resulting in a cytokine storm that is associated with a worse prognosis of infected patients. During aging and obesity, the production of pro-inflammatory cytokines and the establishment of low-grade systemic inflammation are also observed. The expression of components of the renin-angiotensin-aldosterone system, such as ACE2, is also modified by aging and obesity, which could explain why elderly and obese patients are affected and headed the death statistics by COVID-19. Dietary bioactive substances such as polyphenols are able to block the production of cytokines by senescent cells (senescence-associated secretory phenotype; SASP) and adipocytes, as well as modify the ACE-1/ACE-2 ratio, which can potentially result in beneficial effects in COVID-19.

A better understanding of the pathogenesis of SARS-CoV-2 is supported by data generated from previous studies with SARS-CoV and MERS-CoV; however, it is still under construction. Mechanistically, the SARS-CoV-2 virus initially binds to the angiotensin I converting enzyme (ACE)-2 receptor *via* the spike glycoprotein envelope (S-protein) to enter into the target cells (Wan et al., 2020b), a mechanism shared with SARS-CoV (Hofmann et al., 2005) but not by MERS-CoV, that employs dipeptidyl-peptidase 4 (DPP4 or CD26) as a cell entry receptor (Raj et al., 2013). After binding to ACE-2, the virus envelope fuses with membrane epithelial cell, and the RNA strand is released into the cytoplasm of the host cell, initiating viral replication (Figure 1).

The ACE-2 receptor is broad and constitutively expressed in various tissues, such as heart and vascular endothelium (Hamming et al., 2004; Burrell et al., 2005), kidneys (Donoghue et al., 2000), gastrointestinal tract (Hamming et al., 2004), lungs, mainly type II alveolar epithelial cells, and immune cells, including monocytes (mainly in the classical subset of CD14^++^ CD16^−^ cells; Rutkowska-Zapała et al., 2015) and macrophages (mainly in the M1 phenotype; He et al., 2006). ACE-2 is the key enzyme in the balance between the production of angiotensin II (AngII) by the classical pathway of the renin angiotensin system (RAS) and production of angiotensin 1–7 (Ang 1–7) that binds to the orphan MAS receptor (MasR) triggering vasodilator, anti-inflammatory and antifibrotic events, characterizing the “anti-RAS” pathway (Rodrigues Prestes et al., 2017). After entering into the cell, SARS-CoV-2 virus represses ACE-2 expression, which results in an increase of AngII, and exacerbation of inflammation and pulmonary fibrosis (Dalan et al., 2020). The infection of lung epithelial cells and resident immune cells, such as macrophages and dendritic cells results in an important production of pro-inflammatory cytokines that contribute to the worsening of the disease.

In an experimental aging model, it was observed that the expression of pulmonary ACE2 was lower in old- versus young-rats (Xudong et al., 2006). However, when the lung injury was induced with LPS in rats of different ages, there was an imbalance of ACE/ACE2 ratio correlated with strong inflammation, which lead to acute respiratory failure in the age-dependent way (Schouten et al., 2016). A clinical study with patients of different age groups with ARDS demonstrated that the activity of ACE1, ACE2, and the ACE2/ACE1 ratio in bronchoalveolar lavage fluid was no different between groups of neonates, children, adults, or elderly (> 65 years; Schouten et al., 2019). These data indicate that we still need to understand the role of alterations in the ACE2/Angiotensin-(1–7)/MasR axis in the lung of elderly individuals. In the SARS-CoV-2 pandemic’s context, age-dependent decline of ACE2 expression have been associated with COVID-19 fatality (Chen et al., 2020a; Cristiani et al., 2020).

## Cytokine Storm in Covid-19

Cytokine storm syndrome (CSS) is a systemic inflammatory response induced by a wide range of cytokines, resulting in clinical manifestations, such as high fever, lymphadenopathy, hepatosplenomegaly, hyperferritinaemia, and cytopaenia and, if untreated, may progress to multiple organ failure and death (Behrens and Koretzky, 2017; Murthy et al., 2019). The formation of cytokine storm is characterized by a feedforward activation of host immune that causes an uncontrollable release of a several cytokines, such as IFN-gamma, tumor necrosis factor (TNF)-*α*, interleukin (IL)-1, IL-6, and IL-18 resulting in immune regulation disorder (Chousterman et al., 2017; Shimabukuro-Vornhagen et al., 2018). The continuous release of these cytokines triggers a loop reaction characterized by hyperactivation of immune cells, including T cells, macrophages, dendritic, and endothelial cells with further excessive cytokine releasing, which in turn, leads to a self-amplifying hyperinflammatory state known as cytokine storm (Crayne et al., 2019).

Immune response to SARS-CoV-2 infections initially consists in an adaptive immune response necessary to control virus propagation and to prevent disease progression. Once the virus gets to the lung tissue, it will initiate an inflammatory response as part of its immunity to combat the infection (Li et al., 2020). There are strong evidences showing that cytokine storms may participate in the pathogenesis of COVID-19 (Chen et al., 2020d; Huang et al., 2020), similar to prior epidemics such as those caused by SARS and MERS (Channappanavar and Perlman, 2017; Mahallawi et al., 2018). Like many other pathogenic microorganisms, SARS-CoV-2 also evolves mechanisms in order to evade the host immune system. The CSS caused by SARS-CoV-2 enhances the invasion and dissemination of the virus by recruiting different immune cells to the lungs, resulting in an aggressive inflammatory response (Figure 1). The rapid onset of spread inflammation in the lungs of patients infected with SARS-CoV-2 could lead to life-threatening respiratory disorders and subsequent death at the severe stage (Xu et al., 2020). Indeed, Wan et al. (2020a) showed a reduction of 47.62% of natural killer (NK) cells in severe COVID-19 patients. Remarkably, the autopsy findings revealed spleen and lymph node atrophy in COVID-19 patients as well as diffused alveolar damage, and macrophages infiltration indicating that macrophages may also play an important role in CSS induced by SARS-CoV-2 (Wei et al., 2020; Yao et al., 2020).

As previously mentioned, mechanistically the binding of the SARS-CoV-2 spike protein to ACE2 host receptor leads to the downregulation of ACE2, which in turn results in excessive secretion of AngII and reduced secretion of vasodilator angiotensins. AngII plays an important role in proinflammatory response through angiotensin receptor 1 (AT1R). This activated pathway further activates nuclear factor kappa B (NF-kB), which stimulates the overexpression of epidermal growth factor receptor (EGFR) ligands and TNF-α (Eguchi et al., 2018). Indeed, higher levels of ACE2 receptors in lung epithelial cells in children and young adults may have a protective effect on severe COVID-19 clinical manifestations. On the other hand, downregulation of ACE2 and unbalanced Ang II/Ang1–7 level during aging can enhance the cytokine storm (Cristiani et al., 2020). In addition, the hyperactivation of both NF-kB and activator of transcription (STAT)3, leads to a hyperinflammatory state mediated by amplification of IL-6, resulting in increased pulmonary vascular permeability (Murakami et al., 2019). The IL-6 is one of the major cytokines involved in acute inflammation (Scheller and Rose-John, 2006) and was already found to be significantly elevated in severe COVID-19 patients (Chen et al., 2020c; Wan et al., 2020a).

Similarly, the cytokine storm caused by unbalanced AngII/Ang1–7 may also explain the direct cardiovascular system injury of SARS-CoV-2 infected patients. Endothelial dysfunction can increase prothrombotic blood activity and myocarditis, which contributes to the high mortality rate observed in COVID-19 patients (Shi et al., 2020a). Moreover, the virus-induced CSS associated with an unbalanced AngII/Ang1–7 in kidney tubules and podocytes is pointed as responsible for acute kidney injury (Ahmadian et al., 2020). The multiple organ injuries characterized by a high incidence of liver dysfunction, gastrointestinal, and neurological injuries, endocrine alterations, and cutaneous manifestation have also been observed in non-surviving patients (D’Errico et al., 2020).

The cytokine storm landscape of COVID-19 patients was further demonstrated in a retrospective study showing higher concentrations of IL-2, IL-7, IL-10, G-CSF, C-X-C motif chemokine ligand (CXCL)-10, C-C motif chemokine ligand (CCL)-2, CCL-3, and TNF-*α* in the plasma of severe COVID-19 patients (Huang et al., 2020; Lu et al., 2020). Similarly, previous studies also showed higher levels of some cytokines, such as IFN-*γ*, TGF-*β*, IL-1, IL-6, IL-8, and IL-12 in the serum of SARS and MERS patients, highlighting the cytokine storm role in the pathogenesis of severe coronaviruses infection (Channappanavar and Perlman, 2017). Thus, the severity and pathogenicity of the viral infection could be directly correlated to the CSS, which implies that the management of hyperinflammation, the major cause of COVID-19 deaths, would significantly avoid fatal complications. Although there is no standard diagnosis recognition of CSS in COVID-19, it has been proposed that a sudden or rapid disease progression with multiple organ involvement, a significant decline of peripheral blood lymphocyte counts, and an increase of multiple cytokines, such as IL-1β, IL-2R, IL-6, IFN-c, CXCL-10, CCL-2, CCL-3, and TNF-α are the main biomarkers of CSS in COVID-19 patients (Gao et al., 2020).

Additionally, it has been proposed that overactivation of NOD-, LRR-, and pyrin domain-containing protein 3 (NLRP3) inflammasome also has a central as a trigger of cytokine storm. Mechanistically, the multiprotein complexes form in the cytosol and drive caspase-1 cleavage and the secretion of the pro-inflammatory cytokines IL-1β and IL-18 and other DAMPs (Ros et al., 2020). Moreover, it was recently demonstrated that sirtuin 2 (SIRT2) directly represses the NLRP3 inflammasome activity (He et al., 2020). Accordingly, the well reported age-related decline in the activity of the sirtuins (Massudi et al., 2012) might explain age-dependent increases in NLRP3 inflammasome activation (Stout-Delgado et al., 2016).

It has been demonstrated that a viral protein, called viroporin protein 3a leads to a direct activation of NLRP3 in SARS-CoV (Chen et al., 2019). Similarly, the presence of this protein in SARS-CoV-2 genome also suggest a direct activation of NLRP3 (Mousavizadeh and Ghasemi, 2020). Indeed, patients with a reduced immune capacity demonstrated a dysregulated NLRP3 inflammasome activity, which results in severe COVID-19 with tissue damage and a cytokine storm (van den Berg and te Velde, 2020). Considering that NLRP3 is frequently over-activated in elderly individuals, it is believed that the NLRP3 inflammasome plays a central role in the increased lethality observed in aged COVID-19 patients (Lara et al., 2020).

Taking into account that COVID-19 is increasingly being recognized as a syndrome of host inflammatory response, the discovery of effective therapy approaches is urgently needed, especially in certain patients with prior inflammatory-related comorbidities, such as older age, specific genetic background, or obesity, where the CSS promotes the progression to severe organ damage (Shi et al., 2020b). Although glucocorticoid, blood purification therapy, and biological agents, such as interleukins inhibitors may be beneficial to improve the outcome of patients with CSS-induced injury, the efficacy and safety of these approaches still needs to be elucidated in further COVID-19 clinical trials (Qiu et al., 2013; Behrens and Koretzky, 2017; Chousterman et al., 2017; Kogelmann et al., 2017; Norelli et al., 2018; Wang et al., 2020).

As COVID-19 still lacks a specific effective-proven therapy, preventive measures could help to fighting off SARS-CoV-2 infection. It is well known that the decrease of fat mass normalizes the systemic inflammatory status of the body by reducing proinflammatory cytokines. Thus, a diet enriched in functional ingredients that have anti-inflammatory, antioxidant, and immunomodulatory properties should be incorporated in the dietary routine, in particular of those individuals with preexisting hyperinflammatory conditions as obesity or elderly. In this sense, there are some speculative studies about the potential association between vitamin D and the survival of COVID-19 patients, which could be ascribe to its anti-inflammatory properties (Daneshkhah et al., 2020; Grant et al., 2020). It has also been shown that vitamin D has immunomodulatory properties and its deficiency is a risk factor for persistent inflammation and the severe course of COVID-19, which might partly explain the geographic variations of COVID-19 mortality rate (Marik et al., 2020; Rhodes et al., 2020). Another vitamin with potential beneficial role in COVID-19 care management is ascorbic acid. Based on a new clinical trial in Wuhan, China, Carr et al. (Carr, 2020) suggested the potential role of high-dose of ascorbic acid for the treatment and prevention of severe COVID-19. Additionally, several studies have shown that vitamin B3 is highly effective in preventing lung tissue damage (Nagai et al., 1994).

Moreover, it has been suggested that the anti-inflammatory effects of polyphenols may help to overcome COVID-19 severity. Considering the global emergency of this pandemic with regard to the cost and availability of treatment especially in poor countries, it would be also interesting to know how effective polyphenols supplementation is in attenuating cytokine storm in comparison to other agents. It is also important to know at which stage of the COVID-19 the polyphenols supplementation would be the most beneficial. Could they be used in the dietary routine as a prophylactic therapy to prevent cytokine storm at early stages of the disease or would their anti-inflammatory and anti-oxidant properties delay viral dissemination?

## Senescence and Covid-19

Cellular senescence was first reported by Hayflick and Moorehead in 1961 (Hayflick and Moorhead, 1961) as a cellular state characterized by replicative arrest and resistance to apoptosis (Kirkland, 1992). Several intra and extracellular signals can activate molecular pathways, such as cyclin dependent kinase inhibitor 2A (CDKN2A aka p16^INK4a^)-Rb, p53, and CDKN 1A (CDKN1A akap21^CIP1^) to induce senescent cell fate (Beauséjour et al., 2003). Besides of the high level of these key regulators, senescent cells can also present increased lysosomal *β*-galactosidase activity, high DNA damage detected by an accumulation of ƴH2AX, and telomere-associated foci and are usually larger than non-senescent cells (Kirkland and Tchkonia, 2020). Moreover, terminal telomeric repeats shortening after each cell division during the lifespan is also a hallmark of cellular senescence.

Senescent cells accumulate in different tissues during lifespan e.g., in adipose tissue in conditions like diabetes and obesity, in the hippocampi and frontal cortex in Alzheimer’s disease, in the lungs of idiopathic pulmonary fibrosis individuals, in the liver of patients with cirrhosis, and in the kidneys of diabetic kidney disease patients (Kirkland, 2013; Musi et al., 2018; Bian et al., 2019; Justice et al., 2019; Suvakov et al., 2019; Liu and Liu, 2020). Thus, senescence is considered as natural aging process that affects all cell types.

Some senescent cells may also have an hyperinflammatory state caused by secretion of cytokines, chemokines, growth factors, and matrix metalloproteinases, a phenomenon called SASP (Ohtani, 2019). Importantly, cells in SASP can also induce senescence of surrounding cells, and may confer deleterious effects in the tissue microenvironment (Acosta et al., 2013). The aging itself is also associated to the continual production of pro-inflammatory factors, known as “inflammaging” (Sanada et al., 2018; Koelman et al., 2019), which may lead to a chronic inflammation and organ dysfunction. Additionally, other studies suggest that the excess of reactive oxygen species (ROS) production during aging may favor an inflammatory landscape through the increased secretion of pro-inflammatory cytokines, such as TNF-α, IL-1β, IL-2, and IL-6 (Garrido et al., 2019). Interestingly, the excess of pro-inflammatory cytokines can also increase the ROS production, sustaining the inflammaging phenotype (Biswas, 2016).

A key process in immunological aging, also known as immunosenescence, is the decrease of thymic activity in about 99% in elderly people compared to newborns (Gruver et al., 2007), declining the competency of the immune system to combat pathogen infections, such as SARS-CoV-2. Although immunosenescence is described as the progressive loss of all immune effectors, Goronzy et al. (2001) have found a correlation specifically between CD8^+^CD28^null^ T cells and the defective antibody responses to influenza vaccine in elderly adults due to thymic involution. Indeed, the increase in these cells’ population has been consistently observed and is currently used as a biomarker of immunosenescence in older individuals (Zanni et al., 2003). Accordantly, lymphopenia, decrease in CD4^+^ and CD8^+^ T cells population, decrease of B cells and NK cells, monocytes, eosinophils, and basophils are common feature in patients with severe COVID-19. Currently, the available data suggest that the accumulation of senescent T-cells negatively impact the prognosis of COVID-19, as the patients have an ineffective CD8^+^ response, as well as an excessive cytokine secretion from the senescent cells (Cao, 2020; Huang et al., 2020; Qin et al., 2020).

Adipose tissue is a key player in metabolism and inflammation modulation. The adipose tissue dysfunction during aging is likely associated with chronic inflammation (Stout et al., 2017). As age advances, CD38^+^ macrophages and senescent cells accumulate in visceral white adipose tissue producing high levels of inflammatory cytokines in the microenvironment (Covarrubias et al., 2019). Indeed, a recent study reported that obese elderly adults have higher susceptibility to more serious complications of COVID-19 as compared to younger patients. The authors have shown that the mortality rate for these COVID-19 patients was approximately 14% (Petrakis et al., 2020). One possible explanation for this observation could be that the increased secretion of pro-inflammatory cytokines by senescent adipocytes could lead to the cytokine storm in poor prognosis COVID-19 patients.

Although the exact mechanisms of SARS-CoV-2 morbidity and mortality in high risk patients still require extensive research, we may speculate some hypothesis based on the previous SARS-CoV infection understanding, given the high (80%) genetic similarity between both viruses (Yan et al., 2020). The strong correlation between obesity and the disease severity was previously reported in SARS-CoV infected patients. Furthermore, it has been reported that obese patients exhibit delayed and blunted antiviral responses to influenza virus infection, and have poor prognosis (Honce and Schultz-Cherry, 2019). Thus, it has been proposed that obesity may also be an important condition that increases the mortality risk of the SARS-CoV-2 infected patients (Petrakis et al., 2020). Indeed, the Centers for CDC advised that people of any age who have serious underlying medical conditions, including severe obesity [body mass index (BMI) > 40], might be at higher risk for COVID-19 complications and severe illness (Centers for Disease Control and Prevention, 2020).

These findings highlight the importance to look for interventions that remove senescent cells as a preventive treatment strategy against SARS-CoV-2 infection. In this sense, some polyphenols (as quercetin and fisetin) and tyrosine kinase inhibitors (as dasatinib) have been used as senolytic therapy (Yousefzadeh et al., 2018; Hickson et al., 2019). Interestingly, polyphenol-based senolytics alleviate dysfunction in murine models of chronic lung diseases (Schafer et al., 2017), and reduced the mortality of mice infected with mouse β-coronavirus and SARS-CoV-2 viral antigens (Kirkland and Tchkonia, 2020). These findings lead to health regulatory agencies around the world to approve a clinical trial to test flavonoids for elderly hospitalized COVID-19 patients to prevent progression to cytokine storm and ARDS (Table 1).

**Table 1:** Clinical trials evaluating polyphenols in coronavirus disease 2019 (COVID-19) patients.

Identifier	Study title	Intervention	Status	Primary purpose	Phase study
NCT04400890	Randomized proof-of-concept trial to evaluate the safety and explore the effectiveness of a plant polyphenol for COVID-19	Plant Polyphenol and Vitamin D3	Recruiting	Treatment	Phase 2
NCT04377789	Quercetin on prophylaxis and treatment of COVID-19	Quercetin 500 mg (Prophylaxis) Quercetin 1,000 mg (Treatment)	Recruiting	Prevention	n.a.
NCT04578158	Trial to study the adjuvant benefits of quercetin phytosome in patients with COVID-19	Quercetin 500 mg	Recruiting	Treatment	Phase 2
NCT04468139	The Study of quadruple therapy zinc, quercetin, bromelain, and vitamin C on the clinical outcomes of patients infected with COVID-19	Quercetin (500 mg), bromelain (500 mg), zinc (50 mg), and vitamin c (1,000 mg)	Recruiting	Treatment	Phase 4
NCT04622865	Masitinib combined with Isoquercetin and best supportive care in hospitalized patients with moderate and severe COVID-19	Masitinib, Isoquercetin, and best supportive care	Recruiting	Treatment	Phase 2
NCT04536090	Study of Isoquercetin (IQC-950AN) plus standard of care vs. standard of care only for the treatment of COVID-19	Isoquercetin (IQC-950AN)	Not yet recruiting	Treatment	Phase 2
NCT04404218	The Açaí Berry COVID-19 anti-inflammation trial (ACAI)	1,560 mg/day of Açaí Berry extract	Recruiting	Treatment	Phase 2
NCT04392141	Colchicine plus phenolic monoterpenes to treat COVID-19	Oral administration of Colchicine plus Herbal Phenolic Monoterpene Fractions		Treatment	Phase 2
NCT04542993	Can SARS-CoV-2 viral load and COVID-19 disease severity be reduced by resveratrol-assisted zinc therapy (Reszinate)	Zinc Picolinate (50 mg) and Resveratrol (2 g)	Recruiting	Supportive Care	Phase 2
NCT04507867	Effect of a Nss to reduce complications in patients with COVID-19 and comorbidities in stage III (type 2 DM, SAH, and overweight/obesity with BMI <35)	NSS-1 (Spirulina Maxima 2.5 g), folic acid 5 mg, Glutamine 5 g, Cyanomax Ultra (10 g of powder), ascorbic acid 1 g, zinc 20 mg, selenium 100 mcg, cholecalciferol 2000 IU, resveratrol 200 mg, concentrated omega 3 fatty acids (10 grams of powder), L-Arginine 1.5 g, and magnesium 400 mg	Not yet recruiting	Supportive Care	n.a.
NCT04382040	A Phase II, controlled clinical study designed to evaluate the effect of ArtemiC in patients diagnosed with COVID-19	ArtemiC is a medical spray comprised of Artemisinin (6 mg/ml), Curcumin (20 mg/ml), Frankincense (=Boswellia; 15 mg/ml), and vitamin C (60 mg/ml)	Recruiting	Treatment	Phase 2
NCT04403646	Tannin specific natural extract for COVID-19 infection (TaCOVID)	ARBOX [dry extract of polyphenols (tannins) form quebracho and chestnut 240 mg, B12 vitamin 0.72 μg]	Not yet recruiting	Treatment	n.d.
NCT04410510	P2Et extract in the symptomatic treatment of subjects with COVID-19	P2Et (*Caesalpinia spinosa* extract)	Recruiting	Treatment	Phase 2/3
NCT04446065	Protection of health workers against COVID-19 (HERD)	Previfenon® (patent pending) provides 250 mg EGCG	Not yet recruiting	Prevention	Phase 2/3

n.a., not applicable; n.d., not described.

## Polyphenols as a Protective Approach

Polyphenols are key dietary components in preventing inflammatory comorbidities. Interestingly, several plant-derived compounds, such as polyphenols, have been shown to effectively inhibit RNA viruses. Likewise, Zhang et al. (2020) selected biologically proven anti-SARS or MERS coronavirus natural compounds and undertook molecular docking analysis to predict the possible SARS-CoV-2 therapeutic effects of these herbal extracts. They observed that the polyphenols, such as kaempferol, lignan, and quercetin among the 13 anti-inflammatory and anti-oxidant natural compounds potentially suitable for anti-viral usage. Similarly, the results of Singh et al. (2020) suggested that the polyphenols epigallocatechin gallate (EGCG), theaflavin-3-gallate (TF2a), theaflavin-3'-gallate (TF2b), and theaflavin-3,3'-digallate (TF3) can inhibit viral RNA polymerase and may represent an effective therapy for COVID-19.

Even if the consumption of polyphenols is not enough to guarantee a consistent anti-viral effect, many polyphenols have been identified as senolytic agents, which cause the selective death of senescent cells or regulate inflammmageing and immunosenencence. A panel that includes numerous polyphenols in human and murine senescent fibroblasts demonstrated that fisetin (a flavonoid present in fruits and vegetables, such as strawberry, apple, persimmon, grape, onion, and cucumber) and curcumin, were those with the greatest senolitic activity (Khan et al., 2013). Fisetin treatment in mice with progeroid syndrome revealed a reduction in IL-6 levels, which is mainly produced by adipose tissue (Yousefzadeh et al., 2018). Moreover, it has been shown that quercetin, apigenin, wogonin, and kaempferol inhibited the expression of several SASPs markers, including IL-1*α*, IL-1β, IL-6, IL-8, GM-CSF, CXCL1, monocyte chemoattractant protein-2 (MCP-2), and MMP-3 in senescent fibroblasts model. Considering that apigenin was the most powerful to inhibit IL-6, the *in vivo* approach confirmed that this flavone, found mainly in aromatics as parsley, chamomile, celery, and oregano (Shukla and Gupta, 2010), was able to significantly reduces SASP in the kidneys of aged rats (Lim et al., 2015).

Chronic treatment with resveratrol, found abundantly in the skins of red grapes, wine, peanuts, cocoa, and berries (Burns et al., 2002), in senescent lung fibroblasts (MRC5 fibroblasts) reduced the production of IL-6, IL-8, GROα, and VEGF (Pitozzi et al., 2013). Additionally, it has been demonstrated that senescence markers (e.g., IL-6 production) was counteracted by resveratrol in neuroglial cells (Bigagli et al., 2016), vascular smooth muscle cells (Csiszar et al., 2012). Lastly, olive-derived polyphenols including oleuropein, found at very low level in edible table oil olive (Ben Othman et al., 2008), significantly reduced the senescence in chondrocytes, synovial, and bone cells from osteoarthritic patients, an event that was accompanied by reduced activity of the NF-kB transcription factor and reduced SASP markers, as IL-6, IL-1β, and COX-2 (Varela-Eirín et al., 2020).

To date, there are data showing that mice treated for 18 months with resveratrol presented a significant reduction in the expression of ACE1 and an increase in the expression of ACE2 in the aorta, which translated into an increase in serum levels of Ang (1–7) in parallel with the reduction of AngII (Kim et al., 2018). Similar profile was observed in aged kidneys and was associated with improvement in oxidative stress, inflammation, and renal fibrosis (Jang et al., 2018), suggesting that polyphenols could increase ACE2 expression in aged subjects and that these alterations in the ACE2/Angiotensin- (1–7)/MasR axis have beneficial results. Experimental data also demonstrated that resveratrol has an organ-protection function, protecting myocardium in peritonitis/sepsis model (Shang et al., 2019), intestine, liver, kidney, and lung injuries in a hemorrhagic shock model (Müller et al., 2017). It has also been shown a protective role of curcumin and green tea polyphenols in a multiple organ dysfunction syndrome model (Di Paola et al., 2006; Liu et al., 2016).

Furthermore, the consumption of a diet rich in polyphenols has often been claimed as a powerful aid in the control of inflammatory response associated with obesity. The use of resveratrol has been proven to be protective in obesity models through the activation of sirtuin-1, mimicking the caloric restriction, which delay age-related diseases and to extend life span in mammals (Fischer-Posovszky et al., 2010). Resveratrol also inhibited the activation of NLRP3 inflammasome in liver of diet-induced obesity mice, reducing IL-1, IL-6, and TNF-α production (Yang and Lim, 2014), as well as, reduced NF-kb signaling and IL-6 expression in adipose tissue of monkeys fed with high caloric diet (Jimenez-Gomez et al., 2013). Consumption of *yerba mate*, rich in flavonoids like quercetin and rutin, and phenolic acids like chlorogenic and caffeic acid can control inflammation in obesity models (for review, see Gambero and Ribeiro, 2015). Quercetin monotherapy or combined with resveratrol also showed anti-inflammatory activity in adipose tissue, reducing the IL-6 release (Zhao et al., 2017). A*çai* seeds extract, which is rich in proanthocyanidins, in addition to controlling the production of inflammatory mediators, also reduced the expression of AT1 in the adipose tissue of obese mice (Santos et al., 2020). Altogether, these data highlight the hypothesis that these important bioactive dietary components might have modulatory effects on inflammatory pathways present in aging and obesity, as well as, on markers that are been associated with SARS-Cov-2 infection (Figure 1).

Based on the literature evidence showing that polyphenols might be helpful in protecting the body from the negative effects of the disease, several clinical trials are ongoing to test such hypothesis. Indeed, a Phase 2 randomized double-blind placebo-controlled study aims to explore the effectiveness of a commercial plant polyphenols supplemented with vitamin D3 in a set of 200 mild COVID-19 patients (NCT04400890). Moreover, it has been suggested that quercetin, a well-characterized antioxidant, anti-inflammatory and immunomodulatory compound would be a good option in COVID-19 therapeutics (Solnier and Fladerer, 2020). Currently, there are five clinical trials evaluating the adjuvant benefits of quercetin (alone or in combination) in patients with COVID-19 (Table 1) The antiviral properties of resveratrol have also been shown both *in vitro* and *in vivo* (Marinella, 2020). Thus, a Phase 2 study aiming to evaluate the effects of resveratrol as a means to minimize viral load and severity of resulting COVID-19 disease (NCT04542993) is currently ongoing. In addition, a resveratrol-containing nutritional support system is also under investigation to evaluate its effect in reducing complications and comorbidities in the evolution of patients with COVID-19 (NCT04507867). Considering the protective anti-inflammatory and anti-viral effects of tannins (Ueda et al., 2013), a double-blind, randomized trial will be conducted in 140 COVID-19 patients to study the effect of the treatment with dry extract of tannins + B12 vitamin (NCT04403646). Based on promising unpublished *in vitro* and *in vivo* data, the *Caesalpinia spinosa* standardized polyphenol-rich P2Et extract is currently in a Phase2/3 clinical trial (NCT04410510). The study aims to evaluate the efficacy of the supplement in reducing the hospital stay of COVID-19 patients. A brief summary from the clinical trials including polyphenols is shown in Table 1.

## Conclusion and Perspectives

The COVID-19 pandemic brought to light that changes in the cell physiology determined by senescence and inflammation increase substantially the vulnerability of the elderly population and those with comorbidities such as obesity. Indeed, “inflammaged patients” are particularly susceptible to adverse clinical outcomes during SARS-CoV-2 infection and the treatment is challenging. It has been shown that the changes in the expression of the ACE-2 receptor, the imbalance in the angiotensin 1–7/AngII production which increases cardiovascular risk, as well as the increased production of pro-inflammatory cytokines observed in aging and obesity models, can be reversed or controlled by bioactive substances from dietary sources, such as polyphenols. Thus, the data presented here reinforce the hypothesis that polyphenols could have the potential for their use for senescence and inflammation prevention and, therefore for the treatment/management of patients with viral infections such as SARS-CoV-2. It is hoped that the clinical studies under development can add valuable information about this hypothesis and help reduce suffering and mortality imposed by SARS-CoV-2 infection.

## Author Contributions

The authors contributed equally to the writing and the revision of this article. All authors contributed to the article and approved the submitted version.

### Conflict of Interest

The authors declare that the research was conducted in the absence of any commercial or financial relationships that could be construed as a potential conflict of interest.
